# *p75NTR* Promotes Circadian‐Driven Mineralization During Tooth Development via CK2/PER2 Pathway

**DOI:** 10.1111/cpr.70140

**Published:** 2025-11-03

**Authors:** Manzhu Zhao, Hongyan Yuan, Di Wang, Meng Li, Bo Xie, Xuqiang Zou, Mingjie Lu, Ye Qiu, Jinlin Song

**Affiliations:** ^1^ College of Stomatology, Chongqing Medical University Chongqing China; ^2^ Chongqing Key Laboratory of Oral Diseases Chongqing China; ^3^ Chongqing Municipal Key Laboratory of Oral Biomedical Engineering of Higher Education Chongqing China; ^4^ Chongqing Municipal Health Commission Key Laboratory of Oral Biomedical Engineering Chongqing China; ^5^ Department Orthodontics, Hospital of Stomatology Southwest Medical University Luzhou China

**Keywords:** casein kinase 2, circadian rhythm, *p75* neurotrophin receptor, phosphorylation, tooth development

## Abstract

Circadian rhythm is an essential biological process that synchronises physiological activities with environmental light/dark cycles. However, its regulatory mechanisms in tooth development remain incompletely understood. Here, we investigated the role of the *p75* neurotrophin receptor (*p75NTR*) in circadian rhythm regulation and daily mineralization during tooth development using immunofluorescence, circadian rhythm tracking, and genetic models. Spatiotemporal analysis of rat dental germs revealed that oscillatory expression patterns of *p75NTR* closely aligned with clock genes (*Bmal1*, *Clock*, *Per1*, *Per2*), mineralization‐related factors, and odontogenesis‐related factors. *p75NTR* knockout mice (*p75NTR* ^*ExIII−/−*^) exhibited reduced body weight, lower melatonin levels, delayed incisor eruption, decreased daily mineralization width, and downregulation of core clock genes. Mechanistically, *p75NTR* overexpression in immortalised stem cells from the dental apical papilla (iSCAPs) upregulated casein kinase 2 (CK2) expression, enhanced PER2 phosphorylation, and promoted nuclear p‐PER2 accumulation, while CK2 inhibition partially reversed these effects. In vivo, CK2 inhibition via quinalizarin exacerbated incisor eruption defects in *p75NTR* ^*ExIII−/−*^ mice. These findings demonstrate that *p75NTR* regulates circadian‐driven mineralization and tooth morphogenesis, probably via the CK2/PER2 pathway, providing critical insight into the interplay between the circadian rhythm and dental development.

## Graphical abstract

The diagram illustrates how the circadian rhythm regulates the p75NTR‐CK2‐PER2 axis, driving rhythmic biomineralisation during tooth development.

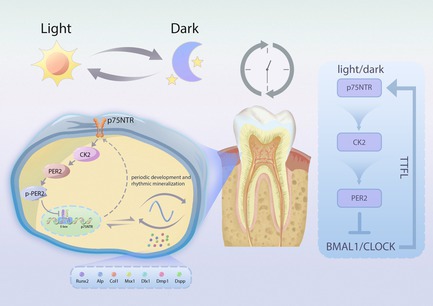

## 1 Introduction

Circadian rhythm, a fundamental biological process mastered by the suprachiasmatic nucleus (SCN) in response to light/dark cycles, plays a crucial role in regulating various physiological functions, such as the sleep–wake cycle, blood pressure, hormone secretion, metabolism, salivary secretion, and tooth development in mammals [1, 2, 3, 4]. The negative transcriptional‐translational feedback loop (TTFL), which involves the core genes *Bmal1*, *Clock*, *Per*, and *Cry* [3, 5, 6, 7], is essential for maintaining the 24‐h rhythm. Evidence indicates that BMAL1, CLOCK, PER, and CRY are expressed at different stages of tooth development and exhibit regular oscillations in a 24‐h cycle [8, 9, 10]. Additionally, several odontogenesis‐related factors, such as ameloblastin, amelogenin, and lysosome‐associated membrane protein, have been reported to display rhythmic expression patterns in rat tooth germs at postnatal day 1 (PN1) [10, 11, 12, 13]. Furthermore, data from incremental growth lines in enamel and dentine further confirm that tooth development exhibits characteristics of the circadian rhythm, which are potentially regulated by clock genes [10, 14, 15].

Recent studies have reported that the CLOCK/BMAL1 complex regulates the *p75* neurotrophin receptor (*p75NTR*) by binding to the E‐box element and identifying *p75NTR* as a novel clock gene regulating oscillatory components in circadian rhythm [16, 17]. As a key maker of cranial neural crest cells, *p75NTR* plays an important role in the epithelial‐mesenchymal interaction during tooth morphogenesis and development [18]. Moreover, *p75NTR* has been reported to be widely expressed in dental germs and can promote mineralization via the Wnt/β‐catenin and phosphoinositide 3‐kinase/Akt signalling pathways during tooth development [19, 20, 21]. Notably, our previous studies demonstrated that *p75NTR* exhibits a 24‐h oscillatory pattern in ectomesenchymal stem cells (EMSCs) [17, 22]. Further, p75NTR‐knockout mice displayed a significantly lower daily mineralization speed and incremental growth line width in the incisors compared to wild‐type mice [23], highlighting the potential role of p75NTR in periodic mineralization and circadian rhythm regulation during tooth development. However, the molecular mechanisms underlying this process remain elusive.

The aim of this study was to further reveal the effect and mechanism of *p75NTR* in the regulation of the circadian rhythm and daily mineralization during tooth development. On the basis of our previous research, which involved in vitro observations of the circadian rhythm dynamics in rat EMSCs, this study focused on in vivo observations of the circadian rhythm dynamics in rat tooth germs. Various molecules and experimental methods, including serological and ELISA analyses of *p75NTR* ^*ExIII−/−*^/*p75NTR* ^*ExIII+/+*^ mice, *p75NTR* overexpression and knockdown experiments, as well as CK2 blocking and rescue procedures, were used to comprehensively explore the mechanism of *p75NTR* in tooth circadian rhythm and periodic mineralization. These findings provide insight into the mechanism underlying circadian rhythm regulation and daily mineralization during tooth development.

## 2 Results

### 2.1 *p75NTR* Exhibited Relative Expression With Clock and Mineralization‐Related Proteins in Dynamic Tissue Immunofluorescence Staining of Tooth Germs

**FIGURE 1:**
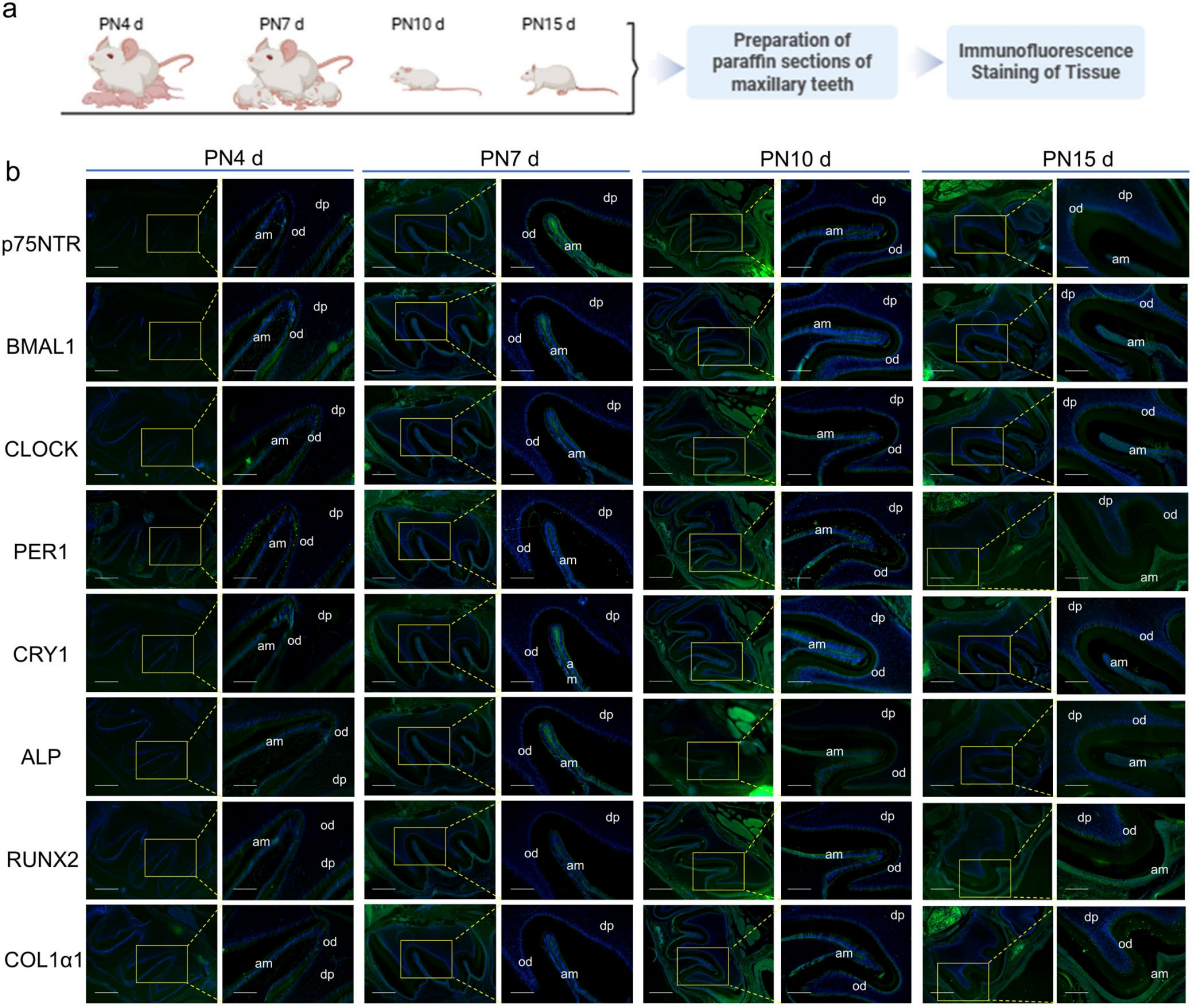
*p75NTR* exhibited relative expression with clock and mineralization‐related proteins in dynamic tissue immunofluorescence staining of tooth germs. (a) Schematic of animal experiments. (b) Images of dynamic histological immunofluorescence staining of newborn rat tooth germs. df: dental follicle; dp: dental papilla; od: odontoblast; am: ameloblast. The scale bars represent 500 μm (left) and 50 μm (right).

The spatiotemporal expression patterns and distribution of p75NTR, clock proteins, and mineralization‐related proteins were investigated during tooth development by tissue immunofluorescence staining of SD rats at postnatal (PND) 4, 7, 11, and 15 (Figure 1a). The results revealed that p75NTR was expressed mainly in the odontoblast and ameloblast layers of the tooth germ at PND4 (Figure 1b). The expression of p75NTR in the ameloblast layer became stronger at PND7 and then weaker at PND10 and PND15. Furthermore, the expression of p75NTR in the odontoblast layer gradually increased from PND7 to PND10 and then PND15. Moreover, p75NTR expression was detected in the hard dental tissues at PND15, implying that p75NTR is closely related to the formation of dentine and enamel.

The core clock proteins (BMAL1, CLOCK, PER1, and CRY1) presented similar expression patterns to that of p75NTR. The difference was that the fluorescence intensity in the hard dental tissue was weak for BMAL1, CLOCK, and PER1. CRY1 was detected in the dental hard tissue at PND10, which was earlier than when p75NTR was detected. The peak expression of p75NTR in the ameloblast layer at PND7 was not observed for the core clock proteins, except for CRY1. The mineralization‐related factors RUNX2, ALP, and COL1α1 were also investigated. The expression pattern of ALP was similar to that of p75NTR, whereas the expression patterns of RUNX2 and COL1α1 were similar to that of CRY1. Together, the above results indicate that p75NTR displays a close relationship with both clock and mineralization‐related factors, and it might participate in the regulation of the circadian rhythm and mineralization during dental hard tissue formation.

### 2.2 *p75NTR*, Mineralization‐Related, and Odontogenesis‐Related Genes Showed a 24‐h Oscillation Pattern Similar to Clock Genes in an in Vivo 48‐h Study of Tooth Germs

**FIGURE 2:**
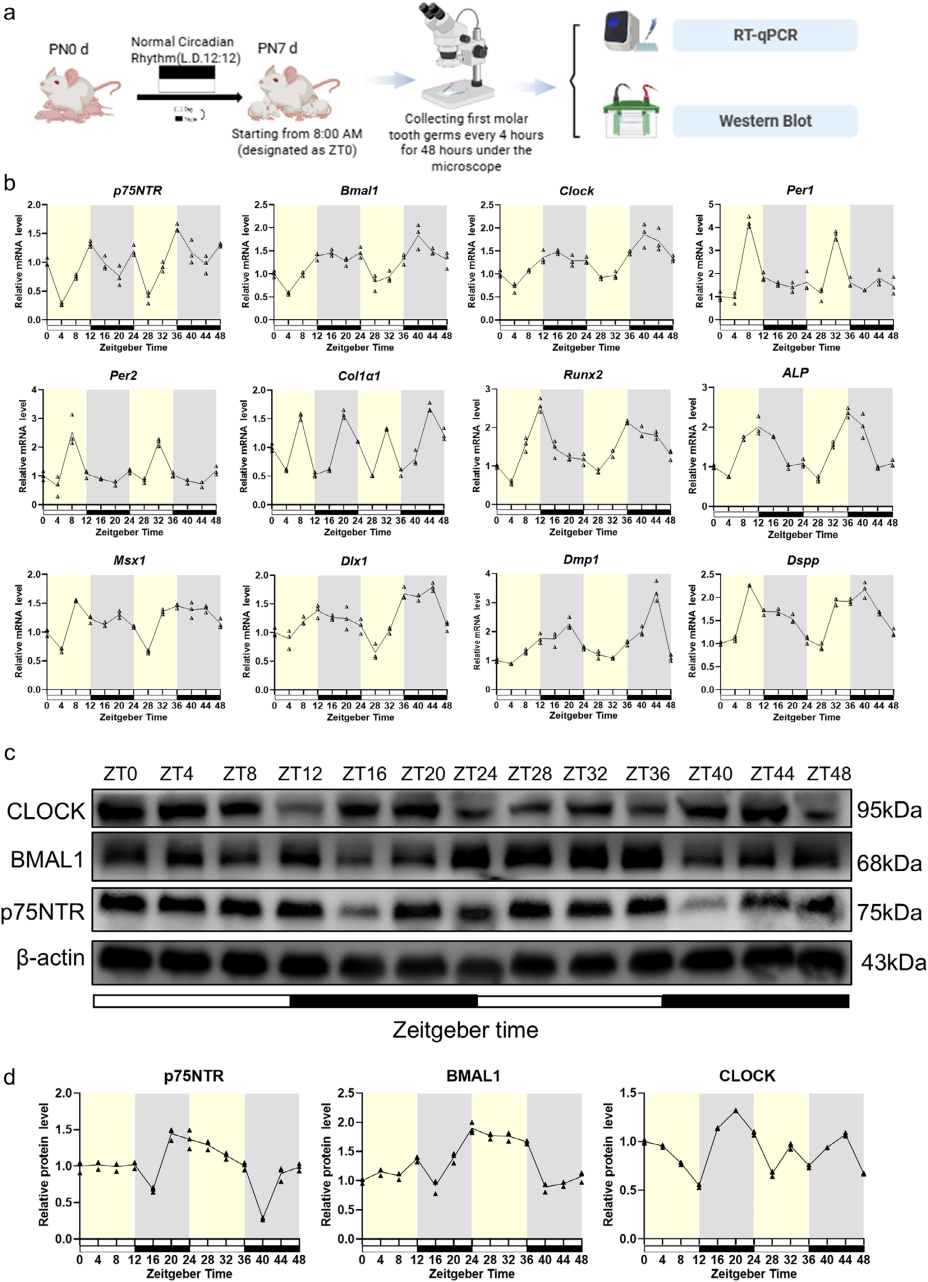
*p75NTR*, mineralization‐related, and odontogenesis‐related genes showed a 24‐h oscillation pattern similar to clock genes in an in vivo 48‐h study of tooth germs. (a) Schematic of animal experiments. (b) qRT‐PCR assessment of the oscillatory expression patterns of *p75NTR*, core clock genes (*Bmal1*, *Clock*, *Per1*, *Per2*), mineralization‐related genes (*Runx2*, *ALP*), and odontogenesis‐related genes (*Msx1*, *Dlx1*, *Dmp1*, *Dspp*). (c, d) Western blot analysis of P75NTR, BMAL1, and CLOCK protein expression dynamics over a 48‐h period. Tissues were sampled at zeitgeber time (ZT) intervals.

In order to gain further insight into the circadian dynamics and functional interplay between p75NTR and core clock components and regulators of mineralization in developing teeth, we carried out qRT‐PCR to analyse the temporal mRNA expression profiles of p75NTR, clock genes, mineralization‐related genes, and odontogenesis‐related genes in PND7 rat tooth germs collected every 4 h from ZT0 to ZT48 (Figure 2a,b). The mRNA expression of *p75NTR* oscillated over an approximately 24‐h cycle and was affected by light stimuli. The expression of *p75NTR* was generally lower in the daytime than at night. Within the *p75NTR* mRNA temporal expression profile, two peaks occurred at ZT4 and ZT28, and four peaks occurred at ZT12, ZT24, ZT36, and ZT48. The four peaks presented two main peaks at light–dark alternating points and two secondary peaks at dark–light alternating points. The dynamic expression of *p75NTR* at the protein level was also examined via western blot. *p75NTR* displayed an approximately 24‐h oscillatory expression pattern (Figure 2c,d), consistent with the results of the qRT‐PCR analysis. However, the two down‐peaks in the western blot analysis occurred at ZT16 and ZT44 at night; both exhibited a 12‐h phase delay compared with the two down‐peaks in the qRT‐PCR analysis (at ZT4 and ZT28 in the daytime); that is, *p75NTR* mRNA expression was lowest in the daytime, whereas p75NTR protein expression was lowest at night. Unlike the four up‐peaks at the mRNA level, there were only two up‐peaks (ZT20 and ZT48) at the protein level. Despite some differences, the data at both the mRNA and protein levels indicate that *p75NTR* expression in rat tooth germs oscillates regularly at approximately 24‐h intervals, which is characteristic of the circadian rhythm.

The core clock genes *Bmal1*, *Clock*, *Per1*, and *Per2* also exhibited approximately 24‐h regular oscillation patterns, similar to *p75NTR* (Figure 2b). The difference was that the up‐peaks exhibited an 8‐h forward phase shift for *Per1* and *Per2*, a 4‐h phase delay for *Bmal1* and *Clock*. The peaks for *Bmal1* and *Clock* occurred at night, whereas those for *Per1* and *Per2* occurred in the daytime. The western blot results revealed that the CLOCK protein expression level exhibited the same trend as the mRNA expression level did (i.e., the peaks occurred at night), whereas the BMAL1 protein expression level exhibited the opposite trend (Figure 2c,d). Together, these data indicate that the expression of the clock genes *Bmal1*, *Clock*, *Per1*, and *Per2* displays a close relationship with p75NTR in rat tooth germs; however, the expression difference suggests a more complex underlying mechanism.

The mineralization‐related factors *Runx2* and *ALP* exhibited an approximately 24‐h regular oscillation pattern, consistent with that observed for *p75NTR* and the clock genes, whereas *Col1α1* exhibited an approximately 12‐h oscillation pattern (Figure 2b). As key factors related to tooth development, *Msx1*, *Dlx1*, *Dmp1*, and *Dspp* were also assessed, with all exhibiting an approximately 24‐h regular oscillation pattern. Like *p75NTR* and the clock genes (*Bmal1*, *Clock*, *Per2*), most of the mineralization‐related and odontogenesis‐related factors were highly expressed at night, implying that hard dental tissue formation is strongly affected by the circadian rhythm and markedly increased during the night. These data might greatly contribute to revealing the reason for the formation of incremental growth lines, for example, daily Retzius' lines in the enamel, von Ebner's lines in the dentine.

### 2.3 *p75NTR* Knockout Decreased the Size of the Body and the Eruption Speed of the Incisors

**FIGURE 3:**
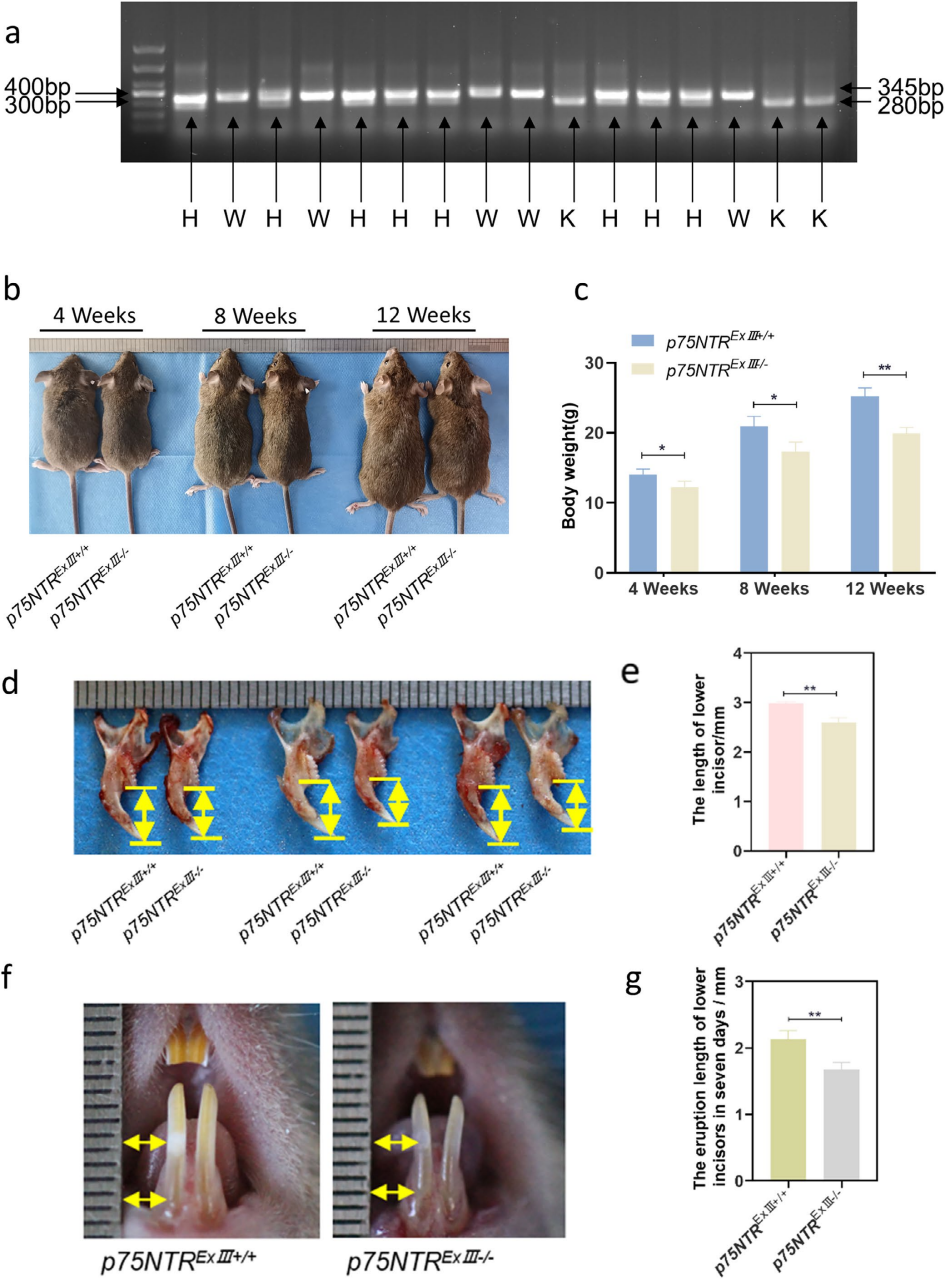
*p75NTR* knockout decreased the size of the body and the eruption speed of the incisors. (a) Genotyping of mice; (b, c) Dynamic body size and weight monitoring across postnatal stages (4, 8, 12 weeks) (*p < 0.05, **p < 0.01); (d–g) Mandibular incisor crown length and eruption speed measurements (**p < 0.01).

To evaluate the effect of *p75NTR* on tooth development, we constructed knock‐out mice and assessed them macroscopically. Firstly, we determined the genotypes of the mice by PCR assays (Figure 3a): eight littermates with two bands at 280 and 345 bp were identified as heterozygous mice (*p75NTR* ^*ExIII+/*^ *−*), five littermates with one band at 345 bp were identified as wild‐type mice (*p75NTR* ^*ExIII+/+*^), and three littermates with one band at 280 bp were identified as knockout mice (*p75NTR* ^*ExIII−/−*^). Dynamic visual observation revealed that the body size of the *p75NTR* ^*ExIII−/−*^ littermates was smaller than that of the *p75NTR* ^*ExIII+/+*^ littermates at three postnatal stages (4, 8, and 12 weeks) (Figure 3b). The body weight of the *p75NTR* ^*ExIII−/−*^ littermates was also lower than that of the *p75NTR* ^*ExIII+/+*^ littermates (*p* < 0.05), although both groups presented a time‐dependent increase in body weight during the observation period (Figure 3c). Moreover, the crown length and eruption speed of the mandibular incisors in *p75NTR* ^*ExIII−/−*^ mice were significantly reduced compared to the *p75NTR* ^*ExIII+/+*^ mice (*p* < 0.01) (Figure 3d–g). The mean mandibular incisor crown length of *p75NTR* ^*ExIII−/−*^ mice (2.60 ± 0.027 mm) was 13.04% shorter than that of *p75NTR* ^*ExIII+/+*^ mice (2.99 ± 0.097 mm). Consistently, the mean eruption speed of *p75NTR* ^*ExIII−/−*^ mice (1.68 ± 0.109 mm/7 day) was 21.13% slower than that of *p75NTR* ^*ExIII+/+*^ mice (2.13 ± 0.126 mm/7 day). These results suggest that p75NTR knockout mice would suppress the size of the body and the eruption speed of the incisors.

### 2.4 *p75NTR* Knockout Inhibited Circadian‐Driven Mineralization in In Vivo Assays of Model Mice

**FIGURE 4:**
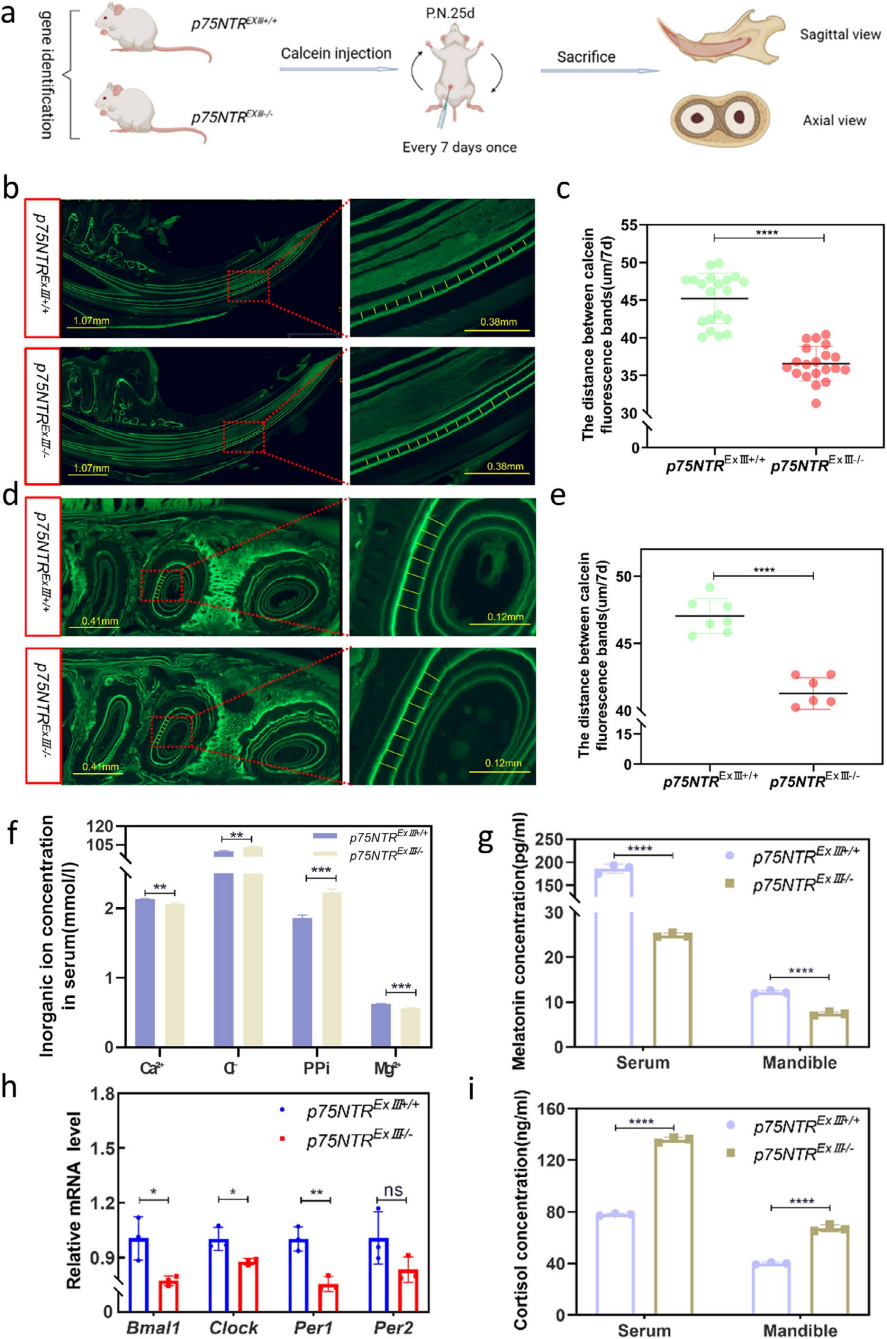
*p75NTR* knockout inhibited circadian‐driven mineralization in in vivo assays of model mice. (a–e) Calcein fluorescence assay to assess mineralization deposition in the incisors and molars (****p < 0.0001). Scale bars: 1.07 mm or 0.41 mm (right), 0.38 mm or 0.12 mm (left); (f, g, i) Serum analysis of ion (Ca^2+^, Mg^2+^, Cl^−^, PPi) and hormone (melatonin, cortisol) levels (*p < 0.05, **p < 0.01, ***p < 0.001, ****p < 0.0001); (h) mRNA quantification of core circadian clock genes (*Bmal1*, *Clock*, *Per1*, *Per2*) (*p < 0.05, **p < 0.01).

To reveal the role of p75NTR in daily mineralization during tooth development, we performed a calcein fluorescence assay (Figure 4a–e). Upright fluorescence microscopy revealed that the distance between the calcein fluorescence bands (representing 7 days of mineralization deposition) was 36.56 ± 2.247 μm for the incisors and 41.27 ± 1.178 μm for the molars in *p75NTR* ^*ExIII−/−*^ mice. These values were significantly lower (incisors: 19.15%; molars: 12.25%) than those in *p75NTR* ^*ExIII+/+*^ mice (45.22 ± 3.254 μm and 47.03 ± 1.302 μm, respectively) (*p* < 0.01). Serological testing revealed that the concentrations of Ca^2+^ and Mg^2+^ in *p75NTR* ^*ExIII−/−*^ mice were significantly lower than those in *p75NTR* ^*ExIII+/+*^ mice (*p* < 0.01), whereas the concentrations of Cl^−^ and PPi were greater (*p* < 0.01) (Figure 4f), highlighting the role of *p75NTR* in promoting mineralization. Meanwhile, the concentration of melatonin, which is synthesised and secreted primarily by the pineal gland in a circadian pattern, was significantly lower in both the serum and mandibles of *p75NTR* ^*ExIII−/−*^ mice than in those of *p75NTR* ^*ExIII+/+*^ mice (*p* < 0.01) (Figure 4g). As a direct negatively correlated partner, the concentration of cortisol was markedly higher in the serum and mandibles of *p75NTR* ^*ExIII−/−*^ mice (*p* < 0.01) (Figure 4i). Moreover, with *p75NTR* knockout in model mice, the expression of the core clock genes *Bmal1*, *Clock*, and *Per1* was dramatically downregulated at the mRNA level in tooth germs(*p* < 0.05) and was positively related to *p75NTR*; the results for *Per2* were similar (Figure 4h). Together, these molecular analyses reveal that tooth daily mineralization is associated with downregulation of core clock genes in *p75NTR* knockout tooth germs, indicating that *p75NTR* might serve a positive role in the rate of circadian‐driven mineralization.

### 2.5 *p75NTR* Promoted Circadian‐Driven Osteogenic and Odontogenic Differentiation in In Vitro Assays at the Cell Level

**FIGURE 5:**
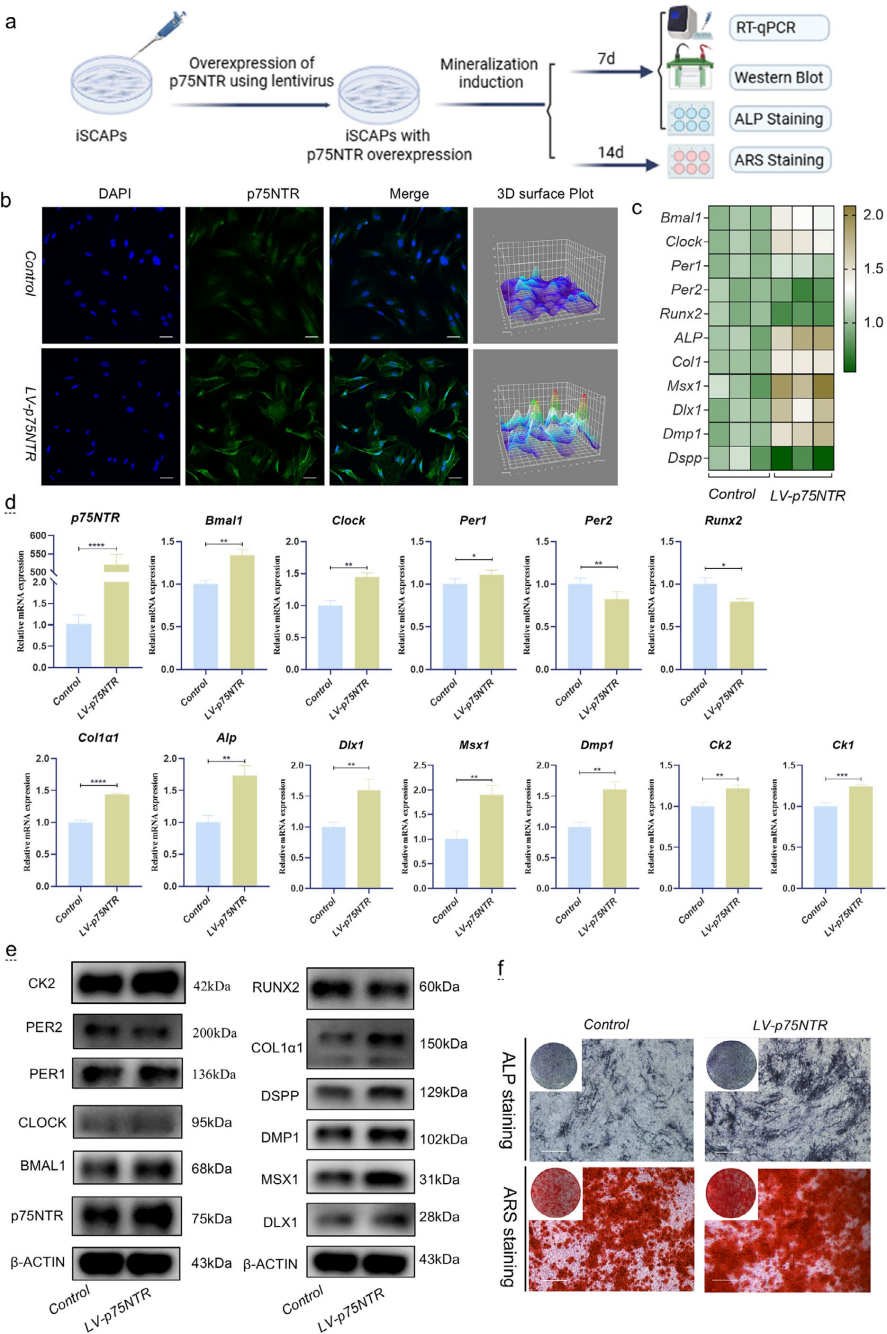
Effects of *p75NTR* Overexpression on the Circadian Rhythm and Mineralization of iSCAPs. (a) Schematic of cell experiments. (b) Immunofluorescence staining confirming p75NTR overexpression (green) in iSCAPs. Scale bars: 50 μm. (c, d) mRNA quantification of clock genes (*Bmal1*, *Clock*, *Per1*, *Per2*), mineralization‐related factors (*ALP*, *Col1α1*, *Runx2*), odontogenesis‐related genes (*Dlx1*, *Msx1*, *Dmp1*), *Ck1*, and *Ck2* (*p < 0.05, **p < 0.01, ***p < 0.001, ****p < 0.0001). (e) Western blot analysis of protein expression. (f) ALP and ARS staining assay for mineralization activity.

Given the spatiotemporal expression and co‐localization patterns of *p75NTR* observed earlier, we utilised lentiviral approaches (without tags) for overexpression and knockdown to investigate the causal link between p75NTR levels and the hierarchy of circadian‐mineralization networks in iSCAPs (Figure 5a). Immunofluorescence assays showed significantly increased green fluorescence intensity in the *LV‐p75NTR* group, with notably sharper peaks in the 3D surface plot (Figure 5b). Furthermore, p75NTR expression in iSCAPs was significantly upregulated, confirming the successful establishment of a p75NTR‐overexpressing cell model, as verified by qRT‐PCR and western blot assays (Figure 5c–e). With *p75NTR* overexpression, the expression levels of most clock factors (*Bmal1*, *Clock*, *Per1*), mineralization‐related factors (*ALP*, *Col1α1*), odontogenesis‐related factors (*Dlx1*, *Msx1*, *Dmp1*), and *Ck2* were dramatically upregulated at the mRNA level (*p* < 0.05) and were positively correlated with *p75NTR* (Figure 5c,d), except for *Per2* and *Runx2*, which displayed the opposite trend. The western blot results were consistent with the qRT‐PCR results, except for those for PER2. In contrast to the findings at the mRNA level, the CK2 protein was significantly positively related to p75NTR. Moreover, the ALP staining and ARS staining intensities were significantly increased in the *LV‐p75NTR* group (Figure 5f), suggesting that *p75NTR* overexpression in iSCAPs significantly promotes the osteogenic ability.

**FIGURE 6:**
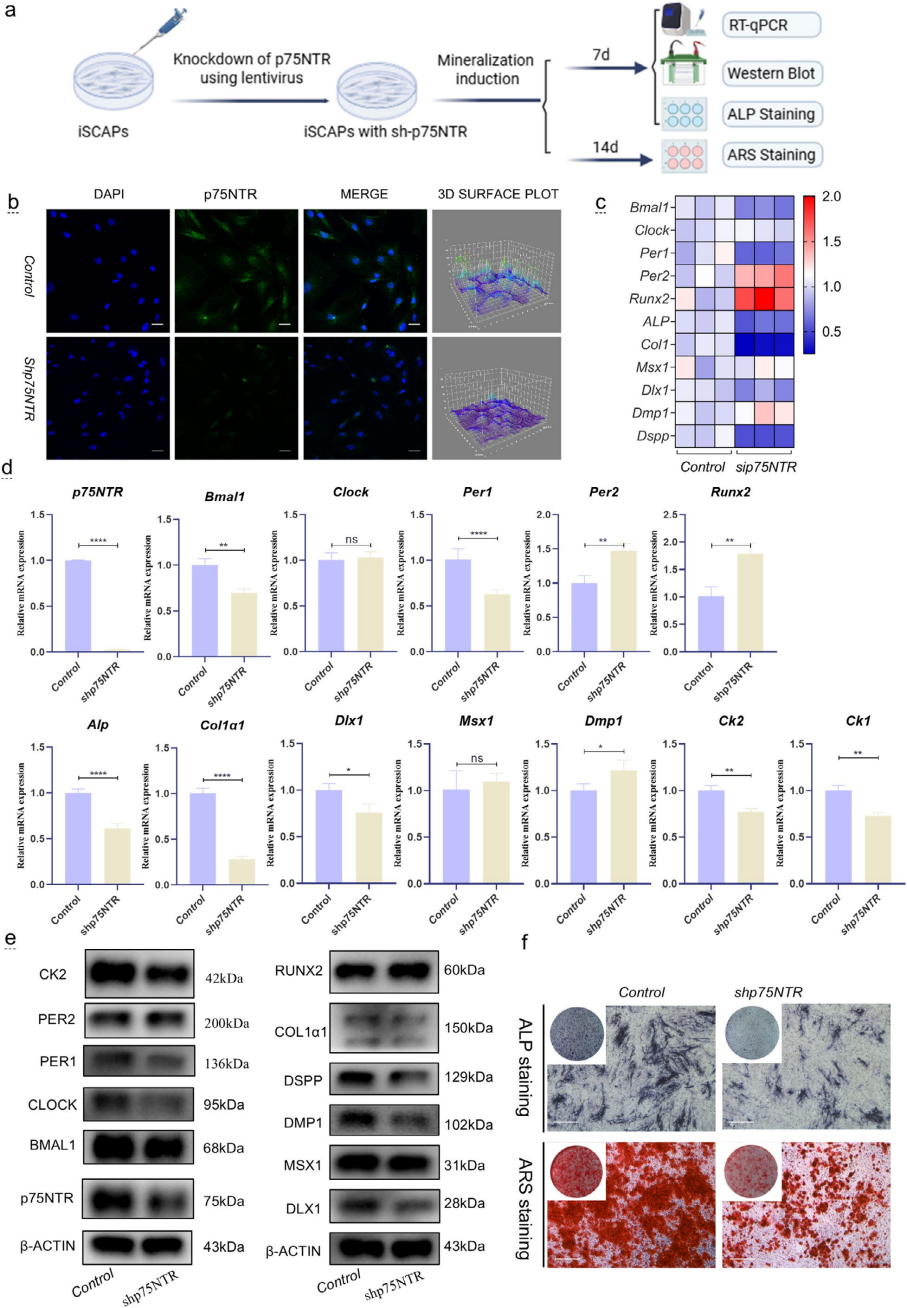
Effects of *p75NTR* Knockdown on the Circadian Rhythm and Mineralization of iSCAPs. (a) Schematic of cell experiments. (b) Immunofluorescence staining of p75NTR (green) in the control and sh‐p75NTR groups. Scale bars: 50 μm. (c, d) mRNA quantification of clock genes (*Bmal1*, *Clock*, *Per1*, *Per2*), mineralization‐related factors (*ALP*, *Col1α1*, *Runx2*), odontogenesis‐related genes (*Dlx1*, *Msx1*, *Dmp1*), *Ck1*, and *Ck2* (*p < 0.05, **p < 0.01, ***p < 0.001, ****p < 0.0001). (e) Western blot analysis of protein levels. (f) ALP and ARS staining assays to assess mineralization activity.

To verify the results of the p75NTR‐overexpressing cell model, we knocked down p75NTR (sh‐p75NTR) in the cell model (Figure 6a). p75NTR expression was successfully downregulated in the *sh‐p75NTR* group (Figure 6b). In the qRT‐PCR assay, the expression levels of the clock genes *Bmal1* and *Per1* showed patterns similar to that observed in the p75NTR‐overexpressing cell model, whereas the expression of *Clock* did not change in the *p75NTR*‐knockdown cell model (compared with the positive relationship observed in the *p75NTR*‐overexpressing cell model) (Figure 6c,d). The mineralization‐related factors *Alp* and *Col1α1* were positively correlated with *p75NTR*, while *Runx2* showed a negative correlation. The odontogenesis‐related factor *Dlx1* exhibited positive relationships, *Dmp1* exhibited negative relationships, and *Msx1* exhibited no changes. Additionally, the expression of *Ck2* mirrored the expression pattern of *p75NTR*. Western blot analysis further confirmed these findings: the clock genes BMAL1, CLOCK, and PER1 were positively correlated with p75NTR, while PER2 showed a slight negative correlation (Figure 6e). The mineralization‐related protein COL1α1 exhibited a slight positive relationship with p75NTR, while RUNX2 demonstrated a clear negative relationship. The odontogenesis‐related proteins DLX1, DMP1, and DSPP were positively related to p75NTR, while MSX1 showed no change. The protein expression of CK2 was consistent with its mRNA expression and positively correlated with p75NTR expression, suggesting that CK2 may be a downstream target of p75NTR. Moreover, ALP and ARS staining further demonstrated that *p75NTR* knockout inhibited the osteogenic ability of iSCAPs (Figure 6f). Together, the p75NTR over‐expression and knockdown cell model results indicate that p75NTR is closely related to clock genes and promotes the mineralization or odontogenesis ability of iSCAPs as a whole; however, the molecular mechanism is unclear.

### 2.6 *p75NTR* Regulated Circadian‐Driven Mineralization via the CK2/PER2 Phosphorylation Axis

**FIGURE 7:**
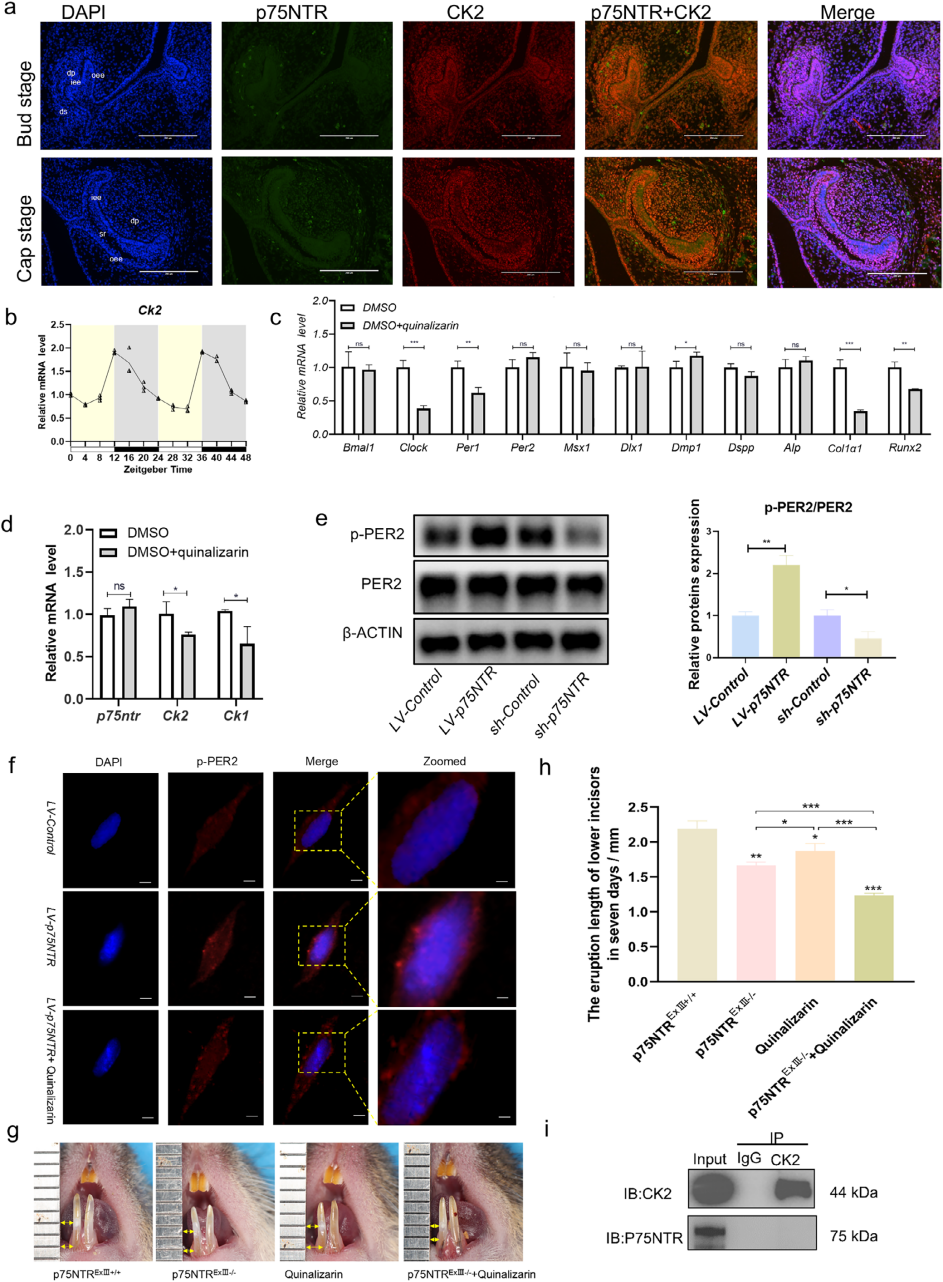
*p75NTR* Regulated Circadian‐driven Mineralization via the CK2/PER2 Phosphorylation Axis. (a) Immunofluorescence staining comparing p75NTR and CK2 spatial expression in tooth germs at the cap and bell stages. Scale bars: 50 μm. (b) qRT‐PCR assessment of the oscillatory expression patterns of *Ck2*. (c) qRT‐PCR analysis of target genes after CK2 inhibition (*p < 0.05). (d) qRT‐PCR analysis of *p75NTR, Ck2* and *Ck1* mRNA after CK2 inhibition (*p < 0.05). (e) The levels of PER2 and phosphorylation assessed by western blot under p75NTR modulation (*p < 0.05, **p < 0.01). (f) Immunofluorescence imaging of p‐PER2 nuclear localization in the *LV‐p75NTR* versus *LV‐Control* groups with/without CK2 inhibition. Scale bars: 20 μm (left) and 5 μm (right). (g, h) In vivo incisor eruption rate measurements following p75NTR knockdown and CK2 inhibition (*p < 0.05, **p < 0.01, ***p < 0.001). (i) Co‐immunoprecipitation (Co‐IP) assay investigating P75NTR‐CK2 protein interaction.

Building on our hypothesis that *p75NTR* coordinates circadian‐mineralization integration via CK2‐dependent signalling, we examined the spatial expression of p75NTR and CK2 during odontogenesis. Immunofluorescence staining at key developmental stages (bud and cap stages) revealed colocalised expression of both proteins (Figure 7a). Additionally, the mRNA expression of *Ck2* in an in vivo 48‐h assay of tooth germs was tested and exhibited an approximately 24‐h regular oscillation which was similar to p75NTR, indicating a potential synergistic spatiotemporal interaction (Figure 7b). To assess the effects of CK2 on the circadian rhythm and mineralization, we further analysed relevant downstream factors following CK2 inhibition. Notably, the circadian genes *Clock* and *Per1* were significantly downregulated (*p* < 0.01), while *Bmal1* and *Per2* levels remained unchanged (*p* > 0.05) (Figure 7c). Regarding mineralization‐related markers, the *Runx2* and *Col1α1* expression levels were significantly decreased (*p* < 0.01), whereas the *ALP* levels showed no significant difference (*p* > 0.05). For odontogenesis‐related factors, Dmp1 was dramatically increased (*p* < 0.05), while *Dlx1*, *Msx1*, and *Dspp* showed no significant changes (*p* > 0.05). Upon overexpression and knockdown of *p75NTR*, a remarkable increase or decrease in *Ck2* and *Ck1* expression, respectively, was observed in iSCAPs (*p* < 0.01) (Figures 5d and 6d). However, blocking CK2 did not result in any significant change in *p75NTR* expression (*p* > 0.05) (Figure 7d), suggesting that CK2 is a downstream target of p75NTR. Additionally, *p75NTR* positively regulated PER2 phosphorylation and its nuclear accumulation. Overexpression of p75NTR increased PER2 phosphorylation, whereas knockdown of p75NTR inhibited PER2 phosphorylation (*p* < 0.01) (Figure 7e). Moreover, the nuclear expression of p‐PER2 was significantly higher in the *LV‐p75NTR* group compared to the *LV‐Control* group (Figure 7f). Collectively, these findings suggest that *p75NTR* functions as an upstream factor of CK2 and PER2 within the signalling network during tooth development, positively regulates PER2 phosphorylation, and promotes the nuclear accumulation of p‐PER2.

To further elucidate the mechanism of *p7NTR* in tooth development, quinalizarin was used to block CK2 in *LV‐p75NTR* cells. The nuclear expression of p‐PER2 was significantly lower in the *LV‐p75NTR*‐quinalizarin group than in the *LV‐p75NTR* group (Figure 7f). This suggests that blocking CK2 can reverse p‐PER2 translocation into the nucleus. Furthermore, in wild‐type mice, blocking CK2 markedly shortened the eruption length of incisors in the quinalizarin group (*p* < 0.01); however, this effect was less pronounced than that observed after knocking out *p75NTR* (Figure 7g). Knocking out *p75NTR* and interfering with CK2 expression resulted in a slower eruption rate of the incisors compared to when either *p7NTR* or CK2 was disrupted alone (*p* < 0.01) (Figure 7g,h). Taken together, the in vitro and in vivo results suggest that *p75NTR* regulates circadian‐driven mineralization during tooth development via the CK2/PER2 phosphorylation axis. However, a Co‐IP assay provided no evidence of direct binding between the p75NTR and CK2 proteins (Figure 7i). These data indicate that *p75NTR*‐CK2‐PER2 participate in the regulation of circadian‐driven mineralization via a non‐canonical pathway during tooth development.

## 3 Discussion

Tooth development involves a sophisticated signalling network triggered by the sequential and reciprocal interactions between the dental epithelium and cranial neural crest‐derived mesenchyme [24, 25, 26]. It is well established that the periodic incremental lines widely present in dental hard tissues indicate that tooth development might be controlled by circadian rhythm [10, 27, 28]. Moreover, recent reports have revealed that the core clock genes and enamel matrix proteins exhibit 24‐h oscillations during odontogenesis [12, 29]. However, further studies are needed to reveal the molecular mechanisms bridging the circadian regulation and biomineralisation. Our study establishes *p75NTR* as a critical mediator of circadian‐driven mineralization through CK2‐dependent PER2 phosphorylation, offering novel insights into the chronobiology of tooth development.

It is believed that the periodic incremental lines widely present in the dental hard tissues indicate that tooth development is controlled by the circadian rhythm [9, 12, 14, 30]. Recently, many studies have reported that clock genes such as *Bmal1*, *Clock*, *Per1*, *Per2*, and *Cry1* are expressed in different‐stage tooth germs during tooth development and exhibit expression oscillations at regular 24‐h intervals [8, 10, 12]. Moreover, several enamel matrix proteins (i.e., ameloblastin, amelogenin, and enamelin) have been reported to exhibit 24‐h oscillation patterns, similar to clock genes, in the tooth germs of PND1 d mice [31, 32]. These data confirm the role of the circadian rhythm in tooth development. However, the exact mechanism remains elusive, and this has severely restricted tooth regeneration approaches to date. In our previous study, *p75NTR*, a key marker of cells derived from the cranial neural crest [18, 33], was shown to be closely related to clock genes, mineralization‐related factors, and odontogenesis‐related factors such as *Bmal1*, *Runx2*, *ALP*, *Msx1*, and *Dmp1* [17, 22]. Here, through spatiotemporal analysis of rat dental germs, we again identified synchronised expression patterns between *p75NTR* and key circadian regulators (*Bmal1*, *Clock*, *Per1/2*), with distinct phase relationships suggesting hierarchical interactions. The observed 4‐h phase advance of *p75NTR* relative to *Per1* and 8‐h delay compared to *Per2* imply potential regulatory cross‐talk within the circadian network. Such dynamics align with recent evidence highlighting that neurotrophin‐mediated phosphorylation of CLOCK‐BMAL1 heterodimers might serve as a mechanism to fine‐tune the circadian amplitude in neural crest derivatives [34, 35]. Notably, *p75NTR* knockout mice exhibited systemic circadian dysfunction characterised by reduced melatonin levels and elevated cortisol, accompanied by delayed incisor eruption and diminished daily mineralization rates. These phenotypic alterations align with the established roles of melatonin in promoting osteogenic differentiation [36, 37, 38] and suggest *p75NTR*'s upstream position in the circadian‐entrained mineralization pathways.

To reveal the exact mechanism of this process, CK2 was investigated, given that it is a known clock kinase that phosphorylates PER2 and regulates p‐PER2 nuclear localization [39, 40, 41]. Moreover, a previous study reported that p75NTR regulates the inflow of depolarized Ca^2+^ via CK2, participating in periodic mineralization [35]. Therefore, CK2 was speculated to be a potential bridge factor in the regulation of periodic mineralization during tooth development by p75NTR. Our results indicated that the expression of CK2 was significantly upregulated or downregulated in iSCAPs in pace with *p75NTR* overexpression and knockdown, respectively, whereas *p75NTR* was not significantly changed when CK2 was blocked. This finding implies that CK2 plays a downstream role in the regulation of p75NTR during tooth development. As a co‐factor of CK2 [42], CK1was detected in this study and showed a similar change pattern to CK2, which further conformed our results. Additionally, we demonstrated that *p75NTR* modulates PER2 dynamics via CK2‐mediated phosphorylation. Overexpression experiments in iSCAPs revealed that p75NTR upregulates CK2 expression, enhances PER2 phosphorylation, and promotes nuclear accumulation of p‐PER2; these effects were partially reversed by CK2 inhibition. While the total PER2 was not changed with p75NTR overexpression and knockdown. The possible reason was that p75NTR might only regulate the PER2 phosphorylation and nuclear accumulation, but not affect the total PER2 expression. These findings are consistent with previous reports, indicating that CK2 phosphorylates PER and promotes its nuclear translocation [43, 44]. This regulatory cascade was further validated in vivo, where pharmacological suppression of CK2 exacerbated incisor eruption defects in *p75NTR* ^*ExIII−/−*^ mice. These data delineate a novel regulatory axis wherein the p75NTR/CK2 signalling cascade dynamically coordinates p‐PER2 subcellular localization through nuclear translocation and CK2‐mediated phosphorylation kinetics, thereby establishing circadian periodicity in odontogenic differentiation. While Co‐IP assays excluded a direct p75NTR‐CK2 interaction, the observed transcriptional regulation suggests the involvement of intermediary factors, requiring future identification. Evidence suggests that p75NTR generates the bioactive lipid ceramide, which directly activates PKCζ, thereby regulating glycogen and lipid synthesis. Additionally, the ceramide‐PKCζ‐CK2 signalling cascade is believed to upregulate antioxidant enzymes through the activation of Nrf2, supporting oxidative phosphorylation [35]. Given the widespread involvement of cellular metabolism, we speculate that p75NTR may regulate the CK2 signalling cascade in tooth development through the ceramide–PKCζ bridge, thereby playing a crucial role in this process.

Intriguingly, mineralization‐related factors exhibited different temporal expression patterns: *Runx2* and *ALP* maintained 24‐h oscillations synchronised with *p75NTR*, while *Col1α1* displayed a 12‐h ultradian rhythm. This temporal segregation may reflect the differential regulation of matrix deposition (predominantly diurnal collagen synthesis) and mineral accumulation (nocturnal mineralization), which is consistent with previous reports on the decoupling of the matrix and mineral phases between day and night [11]. Additionally, the mRNA and protein levels of p75NTR were not fully synchronised. This asynchrony might be attributed to the gene expression complexity from mRNA to protein synthesis involved in post‐transcriptional regulation (e.g., mRNA stability, translational efficiency), post‐translational modifications (e.g., phosphorylation, glycosylation), and differential spatiotemporal expression [45, 46, 47]. Protein synthesis and degradation are tightly regulated within specific cellular compartments, and these dynamics may explain the observed phase shifts [48]. Furthermore, factors such as ribosome availability and translation initiation efficiency could contribute to the disconnect between mRNA and protein expression [49].

Our findings are significant for understanding the formation of incremental lines in dental tissues. The *p75NTR*/CK2/PER2 axis provides a molecular explanation for daily Retzius' and von Ebner's lines, with CK2‐mediated p‐PER2 nuclear translocation potentially serving as the circadian “zeitgeber” for mineralization cycles. These discoveries advance current models of odontogenesis by integrating circadian regulation with established signalling pathways, such as Wnt/β‐catenin and integrin signalling [19, 50, 51], and open new avenues for biomimetic approaches in dental tissue engineering. However, several questions warrant further investigation. First, although this study suggests a potential link between p75NTR and CK2 regulation, the specific intermediary factors remain unclear. It is currently hypothesized that the p75NTR–ceramide–PKCζ–CK2 signalling cascade may exist, but its mechanism and role in tooth development still require further validation. Second, tissue‐specific variations in PER2 phosphorylation dynamics may exist between the dental epithelium and mesenchyme, necessitating lineage‐specific analyses. Finally, a multi‐time‐point dynamic investigation (48 h) would provide a more comprehensive understanding of how p75NTR perturbations affect circadian phase and amplitude; this aspect should be explored in greater depth in the future.

In summary, this study revealed that p75NTR exhibits 24‐h oscillation expression in tooth germs and promotes circadian‐driven mineralization during tooth development. Based on these findings, a new *p75NTR*‐CK2‐PER2 axis is proposed and may serve as the molecular mechanism underlying the regulatory role of p75NTR in the circadian rhythm and daily mineralization of teeth. These findings bridge a key knowledge gap in developmental chronobiology and provide a conceptual framework for the known role of the circadian rhythm in tooth regeneration. However, the signalling networks in tooth development are more complex than initially understood. Further studies are still needed in the future.

## 4 Materials and Methods

### 4.1 Experimental Animals

*P75NTR* ^*ExIII−/−*^ mice were provided by the Jackson Laboratory. These mutant mice exhibited targeted deletion of exon III of the *p75NTR* locus, not deletion of the full‐length *p75NTR*. *P75NTR* ^*ExIII−/−*^(knockout type) and *p75NTR* ^*ExIII+/+*^(wild‐type) mice were identified and used in experiments. Sprague–Dawley (SD) rats were purchased from the Animal Experimental Center of Chongqing Medical University. Animals were housed under specific pathogen‐free (SPF) conditions at 22°C–24°C with 55%–60% humidity, under a 12‐h light/12‐h dark cycle for at least 2 weeks prior to the study, to ensure proper acclimatisation. For anaesthesia, Pentobarbital Sodium was administered via intraperitoneal (IP) injection (Mice: 60 mg/kg; Rats: 35 mg/kg). All animal experiments were carried out with the approval of the Research Ethics Committee of the Affiliated Hospital of Stomatology, Chongqing Medical University (CQHS‐REC‐2023 (LSNo.129)).

### 4.2 Histological Immunofluorescence Staining

SD rats were euthanized at PND 4, 7, 11, and 15 (10%–30% flow rate of CO_2_ per minute chamber volume). The mandibles were dissected and fixed in 4% paraformaldehyde overnight. They were then demineralized with 12% EDTA, dehydrated in a series of graded ethanol solutions, embedded in paraffin, and serially sectioned at 6 μm. The sections were first deparaffinised and prepared using an immunofluorescence detection system kit (Bioss, China) and then subjected to staining. Briefly, the sections were incubated overnight with primary antibodies at 4°C and then incubated with secondary antibodies. Finally, the nuclei were stained with DAPI (Beyotime, China). Images were captured under a fluorescence microscope (Leica, Germany). The antibodies used for incubation are shown in Tables S1 and S2.

Immunofluorescence double staining of p75NTR and CK2 was performed as follows. The mandibles were dissected from E13.5 d to E15.5 d. The procedures before preparation with the immunofluorescence detection system kit (Bioss, China) were the same as those for single immunofluorescence staining. Two heterologous primary antibodies were then diluted and mixed with PBS (rabbit polyclonal antibody against CK2, 1:1000, Novus; mouse monoclonal antibody against P75NTR, 1:250, Abcam), added to the surface of the sections, and incubated at 4°C overnight. The next day, both anti‐rabbit and anti‐mouse antibodies were added, and the sections were incubated for 2 h. The subsequent steps were the same as those for the staining method described above.

### 4.3 Genotype Identification and Somatotype Observation of *p75NTR^ExIII−/−^* Mice

P*75NTR* ^*ExIII−/−*^ and *p75NTR* ^*ExIII+/+*^ littermates were generated by heterozygous breeding (*p75NTR* ^*ExIII+/−*^ and *p75NTR* ^*ExIII+/−*^). PCR‐based genotyping was performed on genomic DNA extracted from tail biopsies to distinguish P*75NTR* ^*ExIII−/−*^, *p75NTR* ^*ExIII+/−*^, and *p75NTR* ^*ExIII+/+*^ offspring using the primer sequences listed in Table S3. For general observations and body weight measurements, male *p75NTR* ^*ExIII−/−*^ and *p75NTR* ^*ExIII+/+*^ mice were anaesthetised for body size recording and body weight measurement at 4, 8, and 12 weeks of age.

### 4.4 Serological Testing of *p75NTR^ExIII−/−^* Mice

Age‐matched *p75NTR* ^*ExIII−/−*^ and *p75NTR* ^*ExIII+/+*^ mice were maintained under standard housing conditions until 8 weeks after birth. Blood samples were collected through the angular vein under isoflurane anaesthesia. After clotting for 2 h at 37°C, the serum was separated by centrifugation at 3000 g for 15 min. Serum concentrations of calcium (Ca^2+^), magnesium (Mg^2+^), chloride (Cl^−^), and inorganic phosphorus (PPi) were quantified using a Rayto Inorganic Ion Detection Kit (Rayto, Shenzhen, China), according to the manufacturer's protocols.

### 4.5 ELISA of Samples From *p75NTR^ExIII−/−^* Mice

Serum and mandibular tissue samples were collected from *p75NTR* ^*ExIII−/−*^ and *p75NTR* ^*ExIII+/+*^ mice. Melatonin and cortisol levels were determined using enzyme‐linked immunosorbent assay (ELISA) kits (Jianglai, China), following the manufacturer's instructions. The serum samples were obtained via the method described above for serological testing. For mandibular tissue processing, the dissected mandibles were rinsed with ice‐cold PBS to remove residual blood, weighed, and mechanically homogenisedhomogenized. The tissue homogenate was prepared in PBS (1:9 w/v) using a glass homogeniser with ultrasonic disruption. After centrifugation at 12,000 rpm (4°C for 10 min), the supernatant was collected for subsequent analysis.

### 4.6 Tooth Germ Extraction From Model Mice

Adult male and female *p75NTR* ^*ExIII+/−*^ mice were housed under standard environmental conditions. On PND7, tail biopsies were performed on newborns for genotyping, followed by stereomicroscopic dissection of the tooth germs. The harvested tooth germ tissues were immediately snap‐frozen in liquid nitrogen. Following genetic confirmation, total RNA was extracted from the tooth germs using a tissue extraction kit for subsequent qRT‐PCR analysis.

### 4.7 Measurement of Mandibular Incisor Length and Eruption Rate

Male *p75NTR* ^*ExIII−/−*^ and *p75NTR* ^*ExIII+/+*^ mice were selected and fed routinely. After euthanasia at 8 weeks of age, the length of the incisor was measured from the tip of the incisor to the mesial surface of the first molar. The rate of incisor eruption was determined by tracking the displacement between the baseline microsurgical mark on the lip enamel surface of the alveolar ridge (8 weeks) and the same anatomical mark (9 weeks) before euthanasia.

### 4.8 Calcein Fluorescence Assay

Male *p75NTR* ^*ExIII−/−*^ and *p75NTR* ^*ExIII+/+*^ mice were maintained on a standard diet for 25 days. Calcein (20 mg/kg) was administered intraperitoneally every 7 days (four injections in total). Animals were euthanized 24 h after the final injection. Dissected mandibles were fixed in 2.5% glutaraldehyde and processed using an EXAKT cutting/grinding system (EXAKT Advanced Technologies GmbH) to prepare 25‐μm sections. Fluorescence microscopy (Olympus IX83; λex = 485 nm/λem = 510 nm) was performed to visualise calcein labelling. Mineralization rates were calculated by measuring the inter‐band distances between successive fluorescent labels using Image‐Pro Plus 6.0 software.

### 4.9 Dynamic Observation of the Circadian Rhythm

Newborn rat littermates were maintained on a standard diet under a 12‐h light/dark cycle (lights on 08:00–20:00, ZT0–ZT12) until PND7. Timed euthanasia was performed at 4‐h intervals commencing at ZT0 (08:00) on PND7: ZT0, 4, 8, 12, 16, 20, 24, 28, 32, 36, 40, 44, and 48. The mandibles were aseptically dissected, with the first molar germs microdissected under stereomicroscopic guidance (Leica Microsystems). The isolated tooth germs were cryopreserved in liquid nitrogen for simultaneous mRNA and protein extraction.

### 4.10 Immunofluorescence Staining

Immortalised dental apical papilla stem cells (iSCAPs) were kindly provided by Prof. Hongmei Zhang (Affiliated Stomatological Hospital, Chongqing Medical University). Immunofluorescence staining of iSCAPs was performed as previously described. Primary/secondary antibody specifications are listed in Tables S1 and S2.

### 4.11 Lentiviral Transfection of *p75NTR*

The *p75NTR* overexpression (*LV‐p75NTR* and control (empty vector)), knockdown (*sh‐p75NTR* and control (empty vector)) virus packages (which used the vector of GV112) were purchased from Shanghai Genechem Co. Ltd. (Genechem, China). iSCAPs were cultivated in six‐well plates with complete medium (DMEM/F12 medium containing 10% FBS and 1% antibiotics) to the appropriate confluence and then transfected with the virus for 12 h at 37°C. The viral transfection supernatant was then changed to complete medium. The successfully transfected cells were harvested and used for subsequent experiments. The specific shRNA sequences and primers used for amplification are listed in Table S4.

### 4.12 Quantitative Real‐Time PCR (qRT‐PCR) and Western Blotting

Total mRNA and protein were extracted and used for the experiments. The qRT‐PCR and western blot assays were performed as previously described [52, 53]. The primer sequences and experimental procedures are shown in Tables S5–S7. The primary and secondary antibodies used are listed in Tables S1 and S2.

### 4.13 ALP Staining and ARS Staining

iSCAPs were seeded into 24‐well plates and cultured with regular culture medium until reaching a confluence of 60%. The medium was then changed to mineralized medium (changed every 3 days). The cells were incubated for 7 or 14 days, and a BCIP/NBT ALP Color Development Kit (Beyotime, China) and ARS staining kit for osteoblast mineralized nodules (Beyotime, China) were used for staining, according to the manufacturer's protocols.

### 4.14 CK2 Inhibition Treatment

iSCAPs (LV‐control group and LV‐p75NTR group) were seeded at a density of 1 × 10^5^ cells/well and treated for 3 h with 50 μM quinalizarin dissolved in dimethyl sulfoxide (DMSO). The other cell samples (LV‐Control and LV‐p75NTR groups) were treated with the same volume of DMSO without the inhibitor. The successfully inhibited cell samples were subjected to qRT‐PCR assays and immunofluorescence staining.

### 4.15 Coimmunoprecipitation (Co‐IP) Assay

Proteins were extracted with protein extraction reagent (Beyotime, China). For the Co‐IP assay, magnetic beads (Bio‐Rad, USA) were first incubated with rabbit polyclonal antibody against CK2 for 2 h, and then the protein lysate was added and incubated at 4°C overnight. The unreacted proteins were separated magnetically and discarded. The remaining steps were the same as those for the western blot analysis described previously.

### 4.16 Statistical Analyses

The data were first analysed via *F* tests to determine the normality of the data distributions. Normally distributed data were analysed by *t* tests, and non‐normally distributed data were analysed by Mann–Whitney tests. The data are expressed as means ± standard errors of the means (SEMs). Statistical significance was assessed via Prism 8.1 software (GraphPad Software, USA). *p* values < 0.05 were considered statistically significant.

## Author Contributions

Manzhu Zhao and Hongyan Yuan carried out the experiments on p75NTR^ExIII−/−^ mice in vivo. Manzhu Zhao, Hongyan Yuan and Di Wang carried out the in vitro experiments on iSCAPs. Manzhu Zhao, Meng Li, Xuqiang Zou and Mingjie Lu conducted the H.E. and immunofluorescence staining. Manzhu Zhao and Hongyan Yuan collected the data. Manzhu Zhao, Bo Xie, Ye Qiu, and Jinlin Song performed the analyses. Manzhu Zhao and Hongyan Yuan wrote the manuscript. Jinlin Song and Ye Qiu supervised and served as administrators of this project. All authors have read and agreed to the published version of the manuscript.

## Ethics Statement

The study was conducted in accordance with the Declaration of Helsinki, and approved by the Research Ethics Committee of the Affiliated Hospital of Stomatology, Chongqing Medical University (CQHS‐REC‐2023 (LSNo.129)).

## Conflicts of Interest

The authors declare no conflicts of interest.

## Supporting information


**Table S1:** Primary antibodies used in this study.
**Table S2:** Secondary antibodies used in this study.
**Table S3:** Genotype identification of *p75NTR* ^*EXIII−/−*^ mice with PCR amplification.
**Table S4:** Primer sequences used in target genes knockdown, and overexpression.
**Table S5:** Specific primers for RT‐PCR of rat samples.
**Table S6:** Mice oligonucleotide primers used in this study.
**Table S7:** qRT‐PCR reaction steps and procedures.

## Data Availability

The data that support the findings of this study are available from the corresponding author upon reasonable request.
