# Supplementary material for: p75NTR Promotes Circadian‐Driven Mineralization During Tooth Development via CK2/PER2 Pathway
Source: Cell Prolif. 2025 Nov 3;59(5):e70140. doi: 10.1111/cpr.70140 (PMC13114795; doi:10.1111/cpr.70140)
Supplement: Supplementary file 1 — Table S1: Primary antibodies used in this study. Table S2: Secondary antibodies used in this study. Table S3: Genotype identification of p75NTR EXIII−/− mice with PCR amplification. Table S4: Primer sequences used in target genes knockdown, and overexpression. Table S5: Specific primers for RT‐PCR of rat samples. Table S6: Mice oligonucleotide primers used in this study. Table S7: qRT‐PCR reaction steps and procedures. [file CPR-59-e70140-s001.docx]

Table S1. Primary Antibodies Used in This Study

| Antibody | Vendor | Catolog/Institution | Clonality |
| --- | --- | --- | --- |
| β-ACTIN | Proteintech | 81115-1-RR | recombinant |
| p75NTR | Abcam | ab245134 | monoclonal |
| CK2 | Novus | NB100-378 | polyclonal |
| BMAL1 | Abcam | ab230822 | monoclonal |
| CLOCK | Abcam | ab3517 | polyclonal |
| PER1 | Bioss | bs-2350R | polyclonal |
| PER2 | Novus | NB100-125 | polyclonal |
| PER2-S662 | Affbiotech | AF4301 | polyclonal |
| CRY1 | Bioss | bs-11441R | polyclonal |
| RUNX2 | Bioss | bs-1134R | polyclonal |
| COL1 | Bioss | bs-0578R | polyclonal |
| ALP | Bioss | bs-2928R | polyclonal |
| MSX1 | Bioss | bs-8512R | polyclonal |
| DLX1 | Bioss | bs-6467R | polyclonal |
| DMP1 | Abclonal | A16832 | polyclonal |
| DSPP | Bioss | bs-10316R | polyclonal |

Table S2. Secondary Antibodies Used in This Study

| Antibody | Vendor | Catolog/Institution | Clonality |
| --- | --- | --- | --- |
| HRP-conjugated Affinipure Goat Anti-Rabbit IgG(H+L) | Proteintech | SA00001-2 | polyclonal |
| HRP-conjugated Affinipure Goat Anti-Mouse IgG(H+L) | Proteintech | SA00001-1 | polyclonal |
| Goat Anti-Rabbit IgG H&L antibody | Bioss | bs-0295G-BF488 | Polyclonal |
| Cy3-labeled Goat Anti-Mouse IgG (H+L) | Beyotime | A0521 | Polyclonal |

Table S3 Genotype identification of *p75NTR^EXⅢ-/-^* mice with PCR amplification

| primers | Upstream (5’–3’) | Downstream (5’–3’) | Product size(bp) |
| --- | --- | --- | --- |
| Primer 1 | TGTTACGTTCTCTGACGTGGTGAG | TCAGCCCAGGGTGTGCACTC | 280 |
| Primer 2 | CTTGGGTGGAGAGGCTATTC | AGGTGAGATGACAGGAGATC | 345 |

Table S4. Primer sequences used in target genes knockdown, and overexpression.

| Primers | Sequence (forward/reverse) | Usage |
| --- | --- | --- |
| *p75NTR#1* | 5'-CGGAGCATATAGACTCCTTTA-3' | Knockdown(shRNA) |
| *p75NTR#2* | 5'-ACAACCTCATTCCTGTCTATT-3' | Knockdown(shRNA) |
| *p75NTR#3* | 5'- GACATGTTCCACAGGCATGTA-3' | Knockdown(shRNA) |
| *p75NTR* | 5'- ATGGATCACAAGGTCTACGC -3' 5'- CCTTATAGTCCTTATCATCGTC-3' | Overexpression |

Table S5. Specific primers for RT-PCR of rat samples

| Genes | Primer sequence | Product size(bp) | NCBI Reference Sequence |
| --- | --- | --- | --- |
| *β-actin* | 5’-ACGGTCAGGTCATCACTATCG-3’ | 155 | NM_031144.3 |
|  | 5’-GGCATAGAGGTCTTTACGGATG-3’ |  |  |
| *p75NTR* | 5’-CCTCATTCCTGTCTATTGCTCCA-3’ | 105 | NM_012610.2 |
|  | 5’-GCGCCTTGTTTATTTTGTTTGC-3’ |  |  |
| *Bmal1* | 5’-CAGAAGCAAACTACAAGCCAAC-3’ | 100 | NM_024362.2 |
|  | 5’-CGGTCACATCCTACGACAAAC-3’ |  |  |
| *Clock* | 5’-CAGTTCTTACAGACATCTCGGTTG-3’ | 89 | NM_001289832.2 |
|  | 5’-AAAGTGCTCTGTTGTAGTGGAAAG-3’ |  |  |
| *Per1* | 5’-ATGCACTTTCAGGCTCCAGT-3’ | 187 | NM_001034125.1 |
|  | 5’-TTGCTTGTATGGCTGCTCTG-3’ |  |  |
| *Per2* | 5’-TGGACTTCGAGGACCTGTTG-3’ | 128 | NM_031678.2 |
|  | 5’-CGATGCTTCTGCTGATGTTG-3’ |  |  |
| *Runx2* | 5’-GAACCAAGAAGGCACAGACAGAA-3’ | 113 | NM_001278483.1 |
|  | 5’-GGCGGGACACCTACTCTCATACT-3’ |  |  |
| *Col1* | 5’-GCCTCAGAAGAACTGGTACATCA-3’ | 108 | NM_053304.1 |
|  | 5’-GAACCTTCGCTTCCATACTCG-3’ |  |  |
| *ALP* | 5’-GCTGAGCAGGAATCCACAAG-3’ | 205 | NM_053304.1 |
|  | 5’-GTGATGAGCGTTAGCGTGTC-3’ |  |  |
| *Msx1* | 5’-CACCCTACGCAAGCACAAGAC-3’ | 139 | NM_031059.2 |
|  | 5’-CGCTCGGCAATAGACAGGTAC-3’ |  |  |
| *Dlx1* | 5’-CAGCCCCTACATCAGTTCCG-3’ | 116 | NM_001100531.1 |
|  | 5’-CTTCTCCGCCTTCCACCAC-3’ |  |  |
| *Dspp* | 5’-GGAAAATGAGCCTCAGGTAGCC-3’ | 198 | NM_012790.3 |
|  | 5’-CCCTGGTTCTCACTCCCCTC-3’ |  |  |
| *Dmp1* | 5’-AAAACAGTGCCCAAGATACCCC-3’ | 124 | NM_203493.4 |
|  | 5’-CCACCTCCTACCCGATATTCCT-3’ |  |  |

Table S6. Mice Oligonucleotide Primers Used in This Study

| Genes | Primer sequence | Product size(bp) | NCBI Reference Sequence |
| --- | --- | --- | --- |
| *β-actin* | 5’-AGATTACTGCTCTGGCTCCTAGC-3’ | 147 | NM_007393.5 |
|  | 5’-ACTCATCGTACTCCTGCTTGCT-3’ |  |  |
| *p75NTR* | 5’-CCAGAGCGAGACCTCATAGC-3’ | 121 | NM_033217.3 |
|  | 5’-AGATGGAGCAATAGACAGGAATG-3’ |  |  |
| *CK2* | 5’-ATCCTGGGACAACATTCACG-3’ | 137 | NM_009974.3 |
|  | 5’-TCTTTGGCGGTCAATCTCTG-3’ |  |  |
| *Bmal1* | 5’-AAGACAATGAGCCAGACAACG-3’ | 147 | NM_001243048.2 |
|  | 5’-TCCCATCTATTGCGTGTCG-3’ |  |  |
| *Clock* | 5’-CCATCCAGTATGCCACAGAAC-3’ | 172 | NM_001289826.1 |
|  | 5’-TCACCACCTGACCCATAAGC-3’ |  |  |
| *Per1* | 5’-CCAGTACAACCAAGCGTAAATG-3’ | 123 | NM_001159367.2 |
|  | 5’-TTGCTGACGACGGATCTTTC-3’ |  |  |
| *Per2* | 5’-AGCGGCTTAGATTCTTTCACTC-3’ | 88 | NM_001420881.1 |
|  | 5’-TCTCATTCTCGTGGTGTTTCC-3’ |  |  |
| *Runx2* | 5’-TTCCAGACCAGCAGCACTCC-3’ | 189 | NM_001420881.1 |
|  | 5’-GCTTCCGTCAGCGTCAACAC-3’ |  |  |
| *Col1* | 5’-TAAGGGTCCCCAATGGTGAGA-3’ | 152 | NM_007742.4 |
|  | 5’-GGGTCCCTCGACTCCTACAT-3’ |  |  |
| *ALP* | 5’-TGACTACCACTCGGGTGAACC-3’ | 94 | NM_001287172.1 |
|  | 5’-TCTGGTGGCATCTCGTTATCC-3’ |  |  |
| *Msx1* | 5’-TCCTCAAGCTGCCAGAAGATG-3’ | 155 | NM_010835.2 |
|  | 5’-CTTGCGGTTGGTCTTGTGC-3’ |  |  |
| *Dlx1* | 5’-ATGCCAGAAAGTCTCAACAGC-3’ | 83 | NM_001416985.1 |
|  | 5’-GAAGGAGACATTTGCTGGTTG-3’ |  |  |
| *Dmp1* | 5’-CAGAGGGACAGGCAAATAGTG-3’ | 168 | NM_001416985.1 |
|  | 5’-CATCGCCAAAGGTATCATCTC-3’ |  |  |
| *Dspp* | 5’-GGACACAGCAGGATAGGTAGCAG-3’ | 124 | NM_010080.3 |
|  | 5’-CACTTTCGTCACTTCCGTTAGATTC-3’ |  |  |
| *Ck1* | 5’-GACTGAAGGCTGCAACAAAGAAA-3’ | 132 | NM_001357498.1 |
|  | 5’-GCAGCCCACGACAGTAATTTAAG-3’ |  |  |

Table S7. qRT-PCR Reaction Steps and Procedures

| Temperature Condition | Time (s) | Cycles/Details |
| --- | --- | --- |
| 95°C | 30 seconds | Initial denaturation |
| 95°C | 5 seconds | Denaturation step |
| 60°C | 30 seconds | Annealing step |
| Repeated for 40 cycles |  |  |
| 65°C → 95°C (Gradually) | 0.5°C increment | Transition step |
| 95°C | 5 seconds | Final denaturation |
| 4°C | ∞ (infinite) | Storage temperature |
